# Supplementary material for: Cloud BioLinux: pre-configured and on-demand bioinformatics computing for the genomics community
Source: BMC Bioinformatics. 2012 Mar 19;13:42. doi: 10.1186/1471-2105-13-42 (PMC3372431; doi:10.1186/1471-2105-13-42)
Supplement: Additional file 1 — Supplementary 1 Cloud BioLinux software documentation in the form of a mini, self-contained website. Users need to download and uncompress the .zip file, and open through a web browser the "index.html" file available on the main directory. (ZIP 1823 kb). [file 1471-2105-13-42-S1.ZIP › Cloud-BioLinux-Package-Documentation/docs/cbdfasta.html]

Bio-Linux Software Documentation Pages

Back to search form

## cbdfasta

|  |  |
| --- | --- |
| Name | cbdfasta |
| Description | **cdbfasta** is one of two platform independent file-based hashing tools released by G. Pertea at TIGR for creating indices for quick retrieval of any particular sequences from large multi-FASTA files. The partner program is called **cdbyank**.  These programs were primarily made available for Bio-Linux as requirements of the Qiime pipeline of programs for processing 16S ribosomal sequences.  If you are interested in indexing fastafiles, we recommend you look at alternatives as well. One worth considering is dbifasta in the EMBOSS suite of tools. |
| Homepage | http://compbio.dfci.harvard.edu/tgi/software/ |
| Remote Documentation |  |
